# Host Antimicrobial Peptides: The Promise of New Treatment Strategies against Tuberculosis

**DOI:** 10.3389/fimmu.2017.01499

**Published:** 2017-11-07

**Authors:** Javier Arranz-Trullén, Lu Lu, David Pulido, Sanjib Bhakta, Ester Boix

**Affiliations:** ^1^Faculty of Biosciences, Department of Biochemistry and Molecular Biology, Universitat Autònoma de Barcelona, Cerdanyola del Vallès, Spain; ^2^Mycobacteria Research Laboratory, Department of Biological Sciences, Institute of Structural and Molecular Biology, Birkbeck University of London, London, United Kingdom

**Keywords:** antimicrobial peptides, innate immunity, tuberculosis, infectious diseases, mycobacteria, antimicrobial resistance, host defense

## Abstract

Tuberculosis (TB) continues to be a devastating infectious disease and remerges as a global health emergency due to an alarming rise of antimicrobial resistance to its treatment. Despite of the serious effort that has been applied to develop effective antitubercular chemotherapies, the potential of antimicrobial peptides (AMPs) remains underexploited. A large amount of literature is now accessible on the AMP mechanisms of action against a diversity of pathogens; nevertheless, research on their activity on mycobacteria is still scarce. In particular, there is an urgent need to integrate all available interdisciplinary strategies to eradicate extensively drug-resistant *Mycobacterium tuberculosis* strains. In this context, we should not underestimate our endogenous antimicrobial proteins and peptides as ancient players of the human host defense system. We are confident that novel antibiotics based on human AMPs displaying a rapid and multifaceted mechanism, with reduced toxicity, should significantly contribute to reverse the tide of antimycobacterial drug resistance. In this review, we have provided an up to date perspective of the current research on AMPs to be applied in the fight against TB. A better understanding on the mechanisms of action of human endogenous peptides should ensure the basis for the best guided design of novel antitubercular chemotherapeutics.

## Introduction

Tuberculosis (TB) is currently one of the most devastating infectious diseases having caused around 1.8 million human deaths, with 10.4 million new cases reported in 2016 and approximately a third of the world’s population harboring its persistent form of the disease-causing pathogen, *Mycobacterium tuberculosis* (Mtb) (1). Statistical analysis of epidemiological data have been shown a steady increase of the disease incidences over the past decade and new drug-resistant forms of TB cases are currently more than 5% of the total. TB has represented a major challenge worldwide and is the first/top leading cause of death from a single infectious microorganism (1).

Although the TB causing pathogen was first identified at the end of the nineteenth century, effective drugs against Mtb were only introduced during the second half of the twentieth century XXs: streptomycin first, followed by isoniazid (INH), pyrazinamide (PZA), ethambutol (EMB), and rifampicin (RIF). Unfortunately, the misuse and overuse of antibiotics for human welfare and farming industry have facilitated the emergence of resistant strains (2–4). Multidrug-resistant TB strains (MDR-TB) do not respond to INH and RIF and extensively drug-resistant strains (XDR-TB) display an added resistance to any fluoroquinolone and at-least one of the three second-line injectable drugs, i.e., amikacin, kanamycin, or capreomycin. Although the development of the first combined anti-TB drug-therapy dramatically improved the disease prognosis outcome, the current alarming rise in multidrug resistance is jeopardizing our early attempt to control the disease (5, 6). Moreover, the current WHO approved treatments impair the patient life quality and have an enormous economic cost (2, 7). Mtb being an extremely successful intracellular pathogen, can remain within the host system by keeping the immune responses under control *via* a wide repertoire of escape pathways (8). To complicate matters further, latent tubercle bacilli infections have become a serious global threat because of the challenge in diagnosing them clinically and their regular conversion from dormancy to active infections in immunocompromised circumstances, due to HIV coinfection, immunosuppressive therapies (9, 10) or diabetes mellitus type 2 conditions (11). Although novel drug susceptibility testing methodologies, such as the GeneXpert^®^ MTB/RIF (12, 13) and HT-SPOTi (14), are enabling the early detection of antibiotic-resistant strains, a complete comprehension of the host immune capability and the mode by which Mtb handles/endures/evades the host defenses will be needed to eradicate this infectious disease (15).

Despite the initial underestimation of the properties of antimicrobial peptides (AMPs) and the difficulties encountered in their attempt to reach the market, nowadays, it is widely accepted that AMPs are multifunctional molecules with key contributions in the mammalian host innate defense (2, 3, 16, 17). In addition, due to the evolution of drug resistance among Mtb strains and their rapid spread across the globe, the use of both natural and synthetic AMPs and their combination with conventional drugs (18, 19) are enabling the creation of a new generation of truly promising antibiotics (20–23). As Mtb can survive and replicate within macrophages, novel anti-TB agents should be able to target the intracellularly dwelling mycobacteria without causing any damage to the host. In this review, we will focus on AMPs, either exploited naturally by our immune system or artificially synthesized, as potential therapeutics to overcome and eradicate the pathogen infection. Special attention will be paid to the diverse mechanisms that can mediate the AMPs’ action against TB infection. Finally, we will discuss the advantages, limitations, and challenges of AMPs for its merchandising and clinical use.

## A Unique and Pathogenic Bacteria

Although most mycobacteria (more than 150 species reported to date) are environmental, only a few species can infect both humans and livestock alike. Mtb is an obligate human pathogen with a low mutation rate (24) and no horizontal gene transfer (25). The TB-causing bacilli have coevolved with our civilization over millennia and its indefinite latency periods probably evolved as an adaptation to the sparse geographic distribution of early human settlements. However, our modern one-world globalization might be triggering a worryingly shorter latency in TB (4).

**Figure 1:**
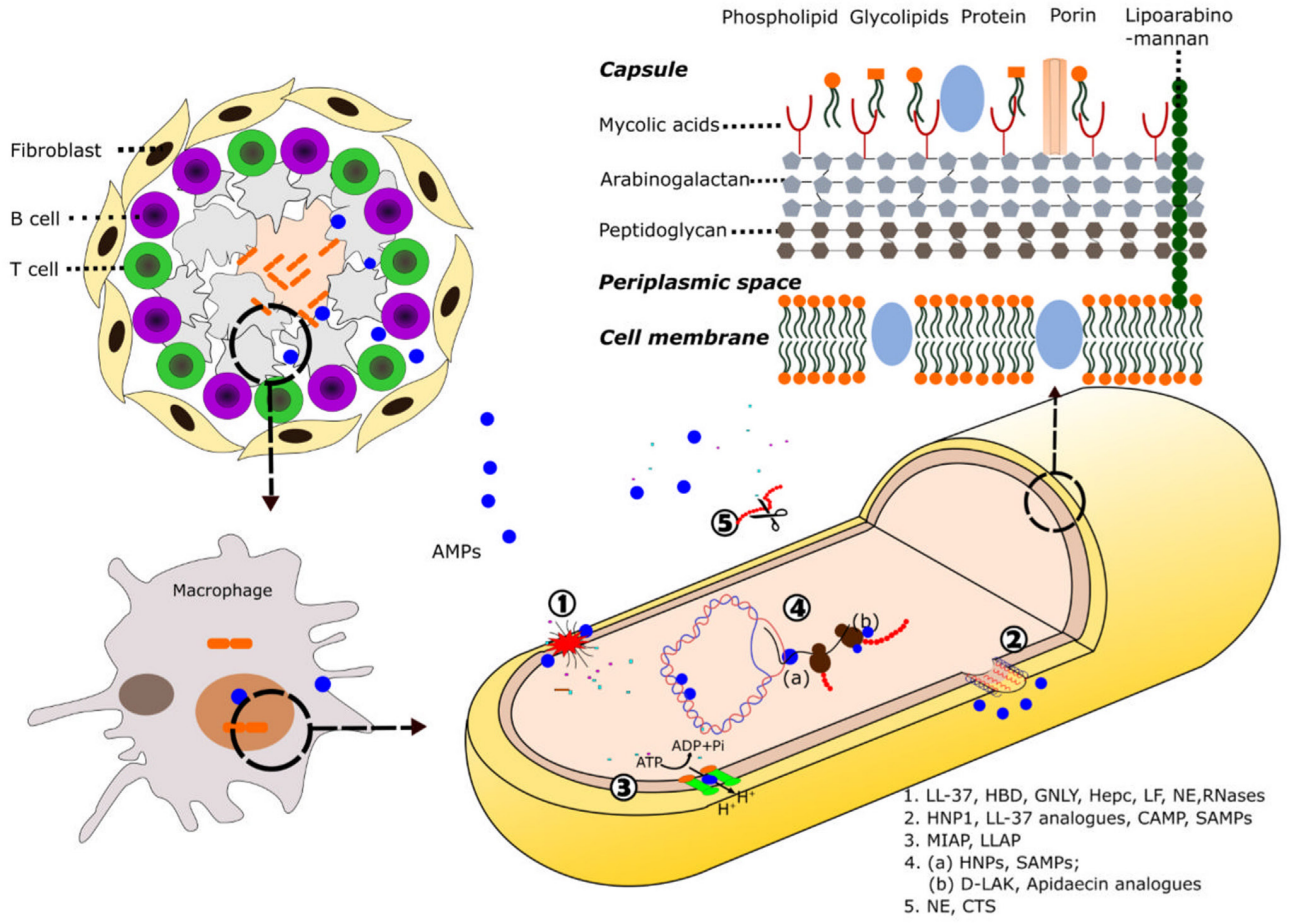
Schematic illustration of AMP mode of action against mycobacteria. Following induction of the immune response by mycobacteria, AMPs are directed toward the area of infection where they can be recruited into the granuloma. At the cellular level, the destruction of the pathogens takes place inside the macrophage phagolysosomes. Composition of the mycobacteria cell wall and the main described mechanisms of action of AMPs against mycobacteria are shown: (1) cell wall and plasmatic membrane disruption, (2) membrane pore formation, (3) inhibition of ATPase, (4) AMP intracellular targets: **(a)** nucleic acids binding, inhibition of replication, and transcription; **(b)** inhibition of translation, and (5) protein degradation. Selected AMPs for each activity are highlighted. See Tables 1 and 2 for a detailed description of each AMP mechanism of action. *Abbreviation*: LL-37, cathelicidin C-terminus; HBD, human β-defensin; GNLY, granulysin; Hepc, hepcidin; LF, lactoferrin; NE, neutrophil elastase; HNPs, human neutrophil proteins; CAMP, cationic antimicrobial peptides; SAMPs, synthetic antimicrobial peptides; MIAP, magainin-I derived antimicrobial peptide; LLAP, LL-37 derived antimicrobial peptide; d-LAK, d-enantiomeric antimicrobial peptides; CTS, cathepsins.

Tuberculosis is mainly an airborne respiratory disease that is conveyed through aerosolized particles. Once in contact with the lung tissues, Mtb can enter and dwell within the host macrophages and other phagocytic immune cells. Immediately after, the infection triggers a complex immune response, and as a result, the pathogens may manage to establish a long-term residence within the host (4, 12, 26). During the primary infection phase, the host defense response sequesters the bacilli in confined cages at the lung alveoli, known as granuloma (Figure 1). During this early period the infected alveolar macrophages, the favorite mycobacterial lodge, are actively releasing pro-inflammatory effectors and other signaling molecules to remove the resident pathogens (8, 27). Following, the tubercle bacilli manage to downregulate the host cell expression profile and enter into a dormant state (26, 28). Ultimately, granuloma will mature and endure a necrosis process. Dormancy responses will facilitate the pathogen’s long-term intra-host survival, and enable it to withstand the necrotic granuloma environment. Upon reactivation of dormant cells, the bacilli will start growing extracellularly and cover the lung cavities with a biofilm layer enriched with the most drug-resistant cells (29). The spread of reinfection is then mediated by coughing induced granuloma mechanical shear (12, 28).

## The Potential of Antimicrobial Peptides in the Anti-TB Chemotherapy: Unraveling Their Mechanisms of Action

Emergence of extensively antimicrobial resistance toward current anti-TB drugs has drawn back our attention toward alternative once neglected therapeutic strategies, including a resurge in AMPs research (2, 30). Expression of endogenous AMPs represents one of the most ancient host defense strategy of living organisms. Their multifunctional mode of action, natural origin, and effectiveness at low concentration have positioned them as prospective candidates in future antitubercular therapeutics market (3, 7, 31, 32). Notwithstanding, to ensure a successful therapy prior to drug design, we must deepen in the knowledge of the underlying mechanism of action of our own innate immunity players.

Despite a low level of amino acid sequence identity, AMPs adopt similar structural folds, indicating the existence of parallel mechanisms of antimicrobial action among distant living organisms (33). Among a significant variety of AMPs traits, we can outline the main common properties. We will review here the main known human AMPs secreted by innate immunity cells to counterbalance mycobacterial infections along with their mode of action.

### Mycobacterial Cell-Wall: A Complex Barrier Particularly Difficult to Overcome

The unusual high antimicrobial resistance in mycobacteria is primarily due to the unique complexity of its cell wall. The complex network of macromolecules such as peptidoglycan, arabinogalactan, and mycolic acids (MAgP complex), which are conglomerated by other proteins and polysaccharides, confirm the main mycobacterial cell-wall scaffold and constitute a highly difficult crossing-barrier for antimicrobial agents (34–37) (Figure 1). The unique covalently-linked MAgP complex of Mtb is a result of mycobacterial adaptation to secure the intracellular survival against continuous selective pressures exerted by the host immune system and other hostile environments. Furthermore, it has been found that the characteristics and composition of the cell wall can be modified during infection (38). The length and structure of the mycolic acids have been related to bacterial intracellular survival and are one of the favorite targets of successful antibiotics (12, 37). Unfortunately, the emergence of Mtb strains with acquired resistance to INH and EMB drugs that target the mycolic acids synthesis, demands novel strategies. Resistant strains have also emerged to PZA, a drug that targets the cell-envelope integrity (2). In this context, dermcidin, a human peptide secreted by sweat glands (39) has been predicted to inhibit the mycolyl transferase enzyme efficiently (40). Other re-emerged research lines target the cell-wall peptidoglycan metabolism (12). On the other hand, one of the main mechanisms by which the AMPs exert their effect is based on the ability to disrupt or permeate the cell membrane (Figure 1), either fully disrupting the lipid bilayer or by creating transient pores (41). Numerous AMPs have acquired amphipathic and cationic structures as short β-sheets and α-helices that allow them to establish interactions with bacterial membranes (42). The first step of AMPs interaction with the pathogen is generally mediated by their positive net charge and hydrophobicity. Unlike eukaryotic cells, in which the anionic lipids are predominantly in the inner leaflet of the lipid membrane providing a neutral cell surface, prokaryotic cells expose a negatively charged surface. Many AMPs can exert a direct killing mechanism against mycobacteria through cell membrane disruption. The binding between the mycobacterial anionic surface compounds and the cationic residues of the peptides promotes the membrane permeabilization (43). Contribution of the peptide cationicity has been corroborated in distinct AMPs by amino acid substitution. As an example, the replacement of lysines by arginines in lactoferrin (LF) variants enhanced their mycobactericidal effect (44). In addition, although the highly hydrophobic scaffold of the mycobacterial envelope offers resistance to AMPs action, the increase in the proportion of α-helical structure and peptide hydrophobicity has being engineered as an alternative strategy to enhance their mycobactericidal features (18). Moreover, some AMPs are directly targeting surface cell-wall proteins to interfere in the cell ion exchange and inhibit the mycobacterial growth. AMPs can interact with the mycobacterial membrane proteins such as ATPases and inhibit the cell pH homeostasis (45, 46). Interestingly, AMPs inducing the membrane permeation can be applied as adjuvants to conventional antibiotics (47).

### Intracellular Targets

Although most of the known AMPs exert their action at the bacterial membrane level, there is a growing number of identified peptides endowed with other previously overlooked targets. Many AMPs have the ability to translocate across the membrane and novel methodologies are bringing the opportunity to identify the peptide interactions with intracellular components (48). As an example, human neutrophil peptides can effectively cross the lipid bilayer without causing significant membrane damage and bind to nucleic acids (49, 50). Selective mycobactericidal action has been achieved by synthetic antimicrobial peptides (SAMPs) that can be internalized by mycobacterial cells and bind to DNA, inhibiting replication, and transcription processes (51). Interestingly, the intracellular action can be achieved at very low peptide concentrations, reducing the potential toxicity to host cells.

### Phagosome-Lysosomal Pathway and Autophagy Modulation

**Figure 2:**
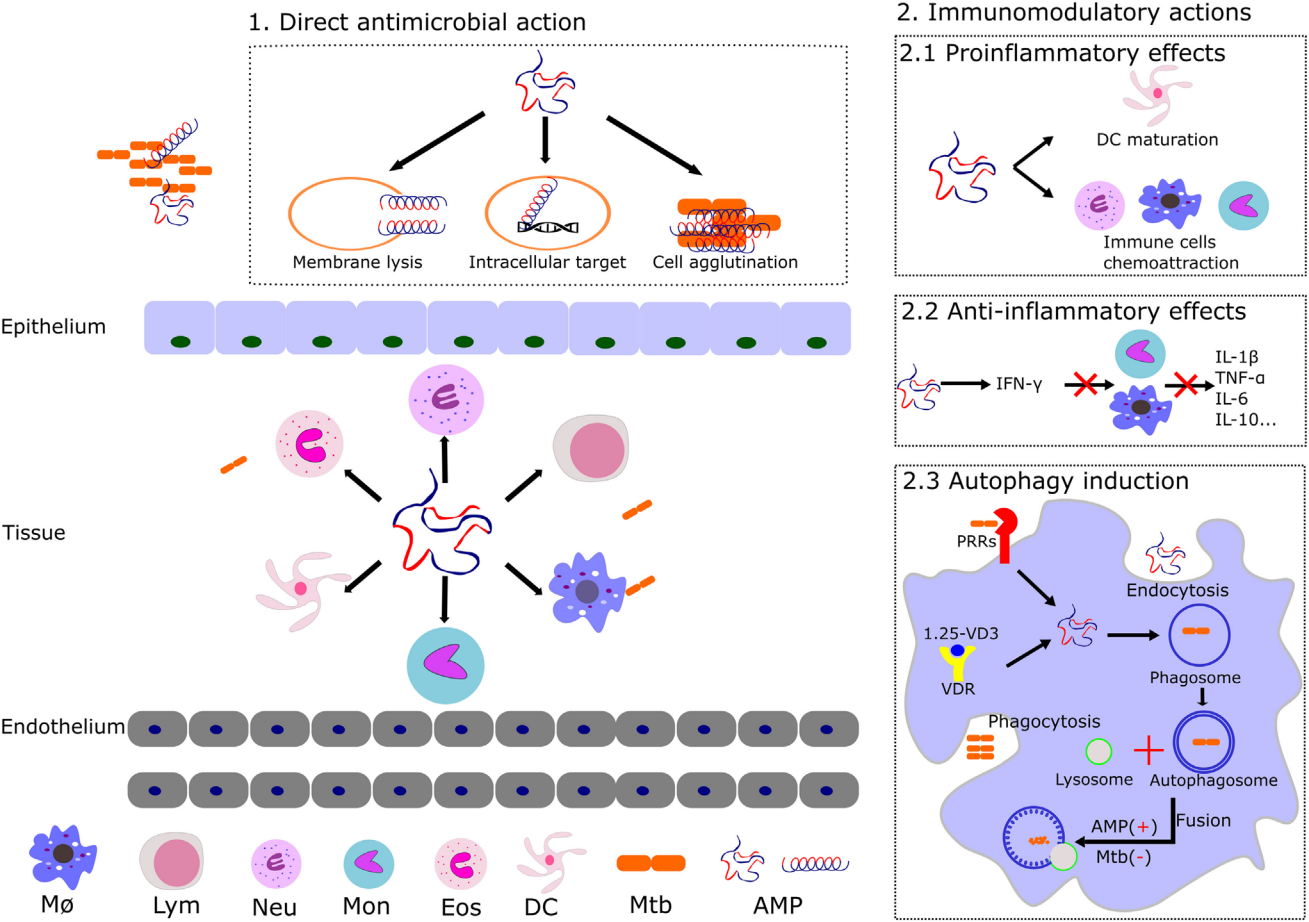
Illustration of the distinct reported mechanism of action of AMPs expressed by the host innate immune cells. The main AMP antimicrobial and immunomodulatory activities are shown: (1) AMPs can trigger the cell lysis, target intracellular key processes (described in Figure 1), and/or agglutinate the bacterial cells. (2) Main AMPs’ immunomodulatory actions that promote the mycobacterial clearance are illustrated. Induction of pro and anti-inflammatory activities contributes to the host defense by regulation of cytokines and chemokines expression and induction of innate cell maturation. AMPs can also intervene in the autophagosome and phagolysosome formation during autophagy. *Abbreviations*: MØ, macrophages; Lym, lymphocytes; Neu, neutrophils; Mon, monocytes; Eos, eosinophils; DC, dendritic cells; Mtb, *Mycobacterium tuberculosis*; AMP, antimicrobial peptides; VD3, vitamin D3; VDR, vitamin D receptor; PRRs, pattern recognition receptors.

*Mycobacterium tuberculosis* has evolved to dwell within one of the most inhospitable cell types, the macrophage. The tubercle bacillus is able to interfere with the phagosomal maturation pathway, blocking the transfer of the phagocytosed compounds to lysosomes (52). At this stage, several mechanisms take place toward the elimination of the pathogen, among them: production of reactive oxygen and nitrogen species, vacuole acidification, lytic enzymes activation, and changes in ion fluxes (53). *Mycobacterium* is able to interfere not only in the recruitment of vesicular ATPase proton pump but also in the acquisition of markers for the endocytic pathway. The TB causing bacilli promote the fusion with early endosomal vesicles but arrest the fusion to the lysosomal compartment, thereby protecting its phagosomal niche from acidification and avoiding the action of lytic enzymes. Moreover, the pathogen inhibits the phosphatidylinositol kinase, reducing the phosphatidyl inositol triphosphate (PIP3) levels and impairing the phagosome maturation (54). The modulation of the phagocytic maturation seems to be carried out by components of the mycobacterial cell wall, such as the mannosylated lipoarabinomannan (7, 54). Altogether, mycobacteria ensure their survival within the host cell by intercepting the autophagic machinery at distinct levels (Figure 2) (8, 55). On their side, many AMPs promoting the phagolysosome formation also contribute to remove the pathogen intruder (56). Thereby, one of the strategy undertaken by the mycobacteria is the downregulation of AMP expression within the macrophage (57). Autophagy has other beneficial effects for the host, such as the restriction of inflammation (58). Indeed, one of main currently used anti-TB drug is rapamycin, an autophagy activator, and the search of novel autophagy inducers is a priority (3, 23, 59).

### Immunomodulatory Activities

Undoubtedly, immunotherapy is at the frontline of TB eradication programs. Following the bacteria engulfment by alveolar macrophages, the mycobacterial components are identified by several pattern recognition receptors resulting in the activation of signaling pathways and the subsequent leukocyte activation (27, 58). In this scenario, participation of endogenous AMPs during the host immune response (Figure 2) is key for a successful eradication of infection (28, 60). We can differentiate two main phases that would mediate the infection process, in the early acute step the AMPs can directly kill the Mtb bacilli, whereas in the secondary late step, the AMPs immunomodulatory action takes the leadership (26). Pro and anti-inflammatory effects can be induced by AMPs mediated by the release of a variety of cytokines (16, 23, 57). Interestingly, the same AMP can have a pro-inflammatory action at an early infection stage, while shifting to anti-inflammatory activity during late infection (3). Indeed, many immune factors play an essential role in the mediation of the infective process (8). For instance, the production of cytokines, which are important for the immune response, such as interferon gamma (IFNγ), are undermined by the mycobacterial infection (61).

## Human Endogenous AMPs Involved in the Fight Against TB Infection

Following mycobacterial infection, a large assortment of antimicrobial peptides is released by our innate immune cells into the affected tissue (62). AMPs as key players of the non-specific immune response (2, 17) have attracted renewed attention as novel therapeutics and several comprehensive databases are now available open to the scientific community (2, 63–65). We describe, here, the main natural human AMPs involved in the fight against TB infection (Table 1).

**Table 1:** Human AMPs involved in immune host defense against mycobacteria.

AMP	Cell type source^b^	Reported activities^a^
Cathelicidin (hCAP18/LL-37)	Neutrophils (66, 67^b^) Monocytes (66, 67^b^) Epithelial cells (66, 67^b^) Mast cells (68) Macrophages (67^b^, 69) Dendritic cells (70) Natural killer cells (71)	Mycobacterial cell wall lysis (72, 73) Immunomodulation (57, 69) Pro-inflammatory action (74)^a^ Autophagy activation (58, 75–78) Chemotaxis (58) Neutrophil extracellular traps (NETs) promotion (73)
Defensins	Eosinophils (HAD) (79)^b^ Macrophages (HBD1) (80) Epithelial cells (HBD1, HBD2, HBD3, HBD4) (7, 81^b^, 82) Dendritic cells (HBD1, HBD2) (80) Neutrophils (HNPs) (7, 30)	Mycobacterial cell membrane lysis (HBD) (23)^a^ (2, 62) Membrane pore formation (HNPs) (7) Mycobacterial growth inhibition (HBD2,3,4) (79, 81, 83^a^, 84) Dendritic and macrophage cells chemotaxis (HBD/HNPs) (82, 85^a^) (23)^a^ Inflammation regulation (HBD) (62, 82) (HNP1) (7, 82) Intracellular DNA target (HNPs) (49, 50)
Hepcidin	Hepatocytes (86) Macrophages (87)^b^ Dendritic cells (88, 89) Lung epithelial cells (89) Lymphocytes (90)	Mycobacterial cell wall lysis (37, 62) Inhibition of mycobacterial infection (91) Iron homeostasis regulation (92, 93) Pro-inflammatory activity (94)
Lactoferrin	Epithelial cells (95) Neutrophils (96) Polymorphonuclear (PMN) leukocytes (97)	Bacterial cell permeation (98) Iron kidnapping (99) Anti-inflammatory activity (100)^a^(101)^a^(102)^a^
Azurocidin	PMN leukocytes (103) Neutrophils (104)	Mycobacterial cell wall lysis (104) Promotion of phagolysosomal fusion (104)
Elastases	Neutrophil azurophilic granules, bone marrow cells (105) Macrophages (106)	Bacterial cell membrane lysis (107) Serine protease activity (108) Cell chemotaxis induction (108) Immunomodulation (109)^a^ NETs formation (110) Macrophage extracellular traps (METs) formation (106, 111)
Antimicrobial RNases	Eosinophils (RNase3/ECP) (79^b^, 112, 113) Neutrophils and monocytes (RNase6) (114) Epithelial cells and leukocytes (RNase7) (115, 116)	Mycobacteria cell wall and membrane lysis (117) Mycobacterial cell agglutination (117)
Eosinophil peroxidase	Eosinophils (118)^b^	Bacterial cell wall lysis (119)
Cathepsins	Neutrophils Monocytes (120)^b^	Mediation of apoptosis pathway (120, 121) Immunomodulation (122)^a^ (109)^a^
Granulysin	Lymphocytes (37)	Mycobacterial cell lysis (37, 123)
Calgranulin/calprotectin	Neutrophils (124, 125) Monocytes (124, 126^b^) Keratinocytes (124, 127) Leukocytes (128)	Phagolysosomal fusion (30, 126) Pro-inflammatory action (125)
Ubiquitinated peptides	Macrophages (37, 129^b^)	Mycobacterial cell lysis (37)
Lipocalin2	Neutrophils (130)^b^	Mycobacterial growth inhibition (37, 130) Immunoregulation (37, 130)

*^a^Reported activities tested in vivo using murine infection models*.

*^b^Reported regulation of AMP expression upon mycobacterial infection*.

### Cathelicidins

Cathelicidins constitute a mammalian family of antimicrobial peptides mostly expressed in leukocytes and epithelial cells in response to different pathogens, contributing to their eradication (7, 37, 72). The human cationic antimicrobial peptide-18 (hCAP-18) is the unique known human member and the leading AMP in TB therapeutics (7, 131). hCAP-18 is essentially conformed by two regions, a highly conserved N-terminal sequence, called cathelin and the bactericidal C-terminal region known as LL-37, released by proteolysis (132, 133). LL-37 contributes to the recruitment of T-cells to the site of infection (66) and displays diverse immunomodulatory and antimicrobial activities (57, 73), undertaking a prominent role during mycobacterial infection (57, 69). In particular, a significant overexpression of LL-37 on neutrophils, epithelial cells, and alveolar macrophages has been observed during Mtb infection (67). The infection of mononuclear cells promotes the upregulation synthesis of LL-37 *via* the vitamin D induction pathway (134). Interestingly, vitamin D deficiency correlates with susceptibility to tuberculosis, while supplementation with vitamin D derivatives improves the efficiency to overcome TB (75). Phagosomal pathway is known to be a key defensive procedure to eradicate Mtb and recent studies point to vitamin D3 as an inducer of autophagy in human monocytes as well as an inhibitor of intracellular mycobacterial growth, *via* upregulation of autophagy-related gene expression (3, 76, 134). The LL-37 peptide thereby decreases, directly or indirectly, the rate of intracellular bacteria proliferation. Recently, transcriptome profiling confirmed the direct contribution of LL-37 at the lysosomal compartment (135). Jointly, all these experimental evidences highlight cathelicidin LL-37 not only as a forthright antimicrobial peptide but also as a prominent modulator of autophagy during mycobacterial infection (3, 77).

### Defensins

Defensins were the first AMPs related to TB by pioneer researchers (49, 50, 81, 136). Defensins are a set of cationic and cysteine-rich peptides with immunomodulatory and microbicidal properties that constitute one of the major and most diverse group of AMPs in the mammalian pulmonary host defense system (3, 16, 137). They are classified according to their structure into alpha, beta, and theta. They show substantial variation in terms of amino acid sequences, and show a diversity of mechanism of action at membrane and intracellular levels. In addition, defensins can be induced and activated by proteolysis pathways to acquire their antibacterial activity (138). Interestingly, high-throughput gene expression of peripheral blood mononuclear cells profile analysis from patients with tuberculosis and Mtb-infected healthy donors revealed the existence of an overexpression of defensins levels in TB patients (139). The peptides were observed to bind to Mtb cells within the macrophage phagosome (140). The essential participation of defensins in the host fight against TB infection has also been corroborated in a murine model (23, 85).

Within the defensin family, we find a variety of cellular source types (Table 1) (82). Noteworthy, the β-defensin2 (HBD2) and the α-defensin (HAD) expression are inducible by mycobacteria wall components in epithelial cells and eosinophils, respectively (79, 81), and could have a preservative role *in vivo* against TB infection. Upregulation of HDB3 and 4 were also reported effective in Mtb MDR-infected mice (83).

### Human Neutrophil Peptides (HNPs)

Human neutrophil peptides are α-defensin type AMPs mainly secreted by neutrophils (50), although low levels of expression have also been detected in monocytes, eosinophils, and epithelial cells (79). HNP-1–4 expression is induced by TB infection (7, 37, 82). On the other hand, although macrophages express only small amounts of HNPs, high intracellular levels can be reached *via* neutrophil-phagocytosis. Interestingly, HNPs have been observed to colocalize with tuberculosis bacilli in early endosomes (84). Moreover, the administration of HNPs maximizes the antimicrobial capacity of macrophages against Mtb (50) and HNP1 was proven effective in a Mtb-infected mouse model (50). HNP-1 can permeabilize the Mtb cell membrane by forming transmembrane pore and then bind to intracellular DNA (7, 50). Interaction with nucleic acids could subsequently inhibit the main cell functions, as transcription and translation (48). On the other hand, combination studies using HNPs and β-defensins with conventional antitubercular drugs have shown a synergistic effect. Therefore, the AMP adjuvant role can reduce the required drug dose and also significantly diminish the bacterial load in vital organs (141). Overall, these findings together with recent experimental work with tuberculosis animal models entrench the therapeutic application in favor of the whole defensin family (7, 37, 83).

### Hepcidin

Hepcidin (Hepc) is a short and highly cationic antimicrobial peptide that was originally detected in serum and urine (142). It adopts a hairpin loop that encompasses two short beta-strands. Hepcidin is predominantly synthesized in hepatocytes and is released from a precursor by proteolysis. Its expression is induced by infectious or inflammatory processes and plays a prominent role in the iron homeostasis, regulating uptake, and mobilization (92, 143). Specifically, hepcidin can downregulate the transmembrane transport of iron through its union with ferroportin, a transmembrane protein that exports iron to the extracellular space (93). The reduction in extracellular iron concentrations makes pathogen invasion conditions more hostile (91). Interestingly, during infection, hepcidin is released into the bloodstream and is considered to be responsible of the anemia associated with inflammation (94). Indeed, anemia is a common difficulty encountered in TB (144). Moreover, Lafuse and coworkers demonstrated that mycobacterial infection induced the emergence of high levels of hepcidin in macrophages phagosomes and confirmed the peptide inhibition of Mtb growth *in vitro* (87). Further research also reported the presence of hepcidin in other innate cell types such as dendritic cells. The peptide expression in non-phagocyte cells suggests an extracellular mycobactericidal activity mediated by iron reduction in both alveolar and interstitial spaces (88). Particularly, due to the hepcidin effect on iron levels, differences in the expression of the peptide could be related to different phenotypes of iron homeostasis in TB patients. A significant correlation was observed between serum hepcidin levels and the promoter polymorphism in TB patients and was suggested to be considered in the diagnosis and prognosis of tuberculosis (145).

### Lactoferrin

Lactoferrin is another AMP related to iron homeostasis regulation. It is a multifunctional iron binding glycoprotein present in several tissues and most human body fluids. It has a molecular weight of 80 kDa and belongs to the transferrin family (99). LF and its natural N-terminal fragment released by proteolytic cleavage (*Lactoferricin*, LFcin) participate in host defense and have wide spectra antimicrobial effects (37, 44, 98). Noteworthy, LF is the only AMP given by systemic administration that is currently in clinical trials (146). Diverse studies have demonstrated the presence of LF in macrophages and blood cells and its activity against *Mycobacterium*. Moreover, LF immunomodulatory capacity can also contribute to the eradication of TB. Particularly, it has been observed that mice treated with LF manifest an increase in the proportion of IL-12/IL-10, which results in increased Th1 cells, with a protective role against Mtb (100, 101). The anti-inflammatory properties of human and mouse LF were also corroborated in another Mtb mouse infected model (102). In addition, other studies clearly demonstrated the immunomodulatory role of LF, improving BCG-vaccine efficacy when used as adjuvant (147, 148). Recently, it has been reported that LF expressed in azurophilic granules of neutrophils is capable of killing *M. smegmatis* (104).

### Lipocalins

Lipocalins are a family of peptides involved in cellular traffic and inflammation which are also related to the iron homeostasis (149). Lipocalin2, also called neutrophil gelatinase associated lipocalin, is expressed in neutrophils and displays anti-TB activity (130).

### Azurocidin

Azurocidin, a leukocyte polymorphonuclear (PMN) granule protein, is a cationic antimicrobial protein of 37 kDa, also called CAP37 or heparin-binding protein, due to its high affinity for heparin (103). Shortly after its discovery it was found that azurocidin, like other antimicrobial proteins, not only displayed an antimicrobial activity but was also capable of exerting a mediating role in the modulation of the host defense system (150). Azurocidin is stored in secretory granules and is released into the endothelial area by PMN cells, rapidly reaching the infected or inflammation area (151). Azurocidin, at the front line of infection, activates monocytes, macrophages, and epithelial cells (152). Moreover, azurocidin has a wide range antimicrobial activity, working efficiently at acidic pH, a condition promoted in mature phagolysosomes (153). Interestingly, it has recently been reported that azurophilic granule proteins are implicated in mycobacterial killing, facilitating the fusion of mycobacteria-containing phagosomes with lysosomes (104).

### Elastases

Elastases are serine proteases secreted by neutrophils and macrophages involved in the fight against pulmonary infections (107). One of the best studied elastase is the neutrophil elastase (NE), also known as elastase2, a 29-kDa protein expressed during myeloid development and secreted by neutrophils during episodes of infection and inflammation (107, 108). NE was reported to confer a protective effect against *M. bovis* in mice pulmonary tract (109). Many studies emphasize NE multi-functionality; the protein can break the tight junctions to facilitate the migration of PMN cells to the inflammation/infection area and induce cell chemotaxis (108). The neutrophil granule protein can work within the macrophage phagosomes (154). Complementarily, NE is also reported to kill mycobacteria extracellularly in a rather peculiar way. Neutrophil granules can release their protein cargo together with chromatin, resulting in the formation of extracellular fibrillar structures that facilitate bacteria arrest. NE colocalizes with the neutrophil extracellular traps and can facilitate the degradation of virulence factors (110, 155). Interestingly, heavily infected macrophages can also explode and form extracellular traps, a process which is also regulated by elastases (106, 111).

### Cathepsin (CTS)

Cathepsin is another serine protease involved in the host defense against TB infection that is mainly expressed in neutrophils and macrophages (130, 156). Procathepsins are converted to the mature enzyme in acidic conditions and are active within the lysosomal compartment (30). The Mtb bacilli can downregulate CTSs expression in macrophages to ensure its intracellular survival (120, 156). The antimicrobial protease is proposed to protect the host mostly by an immunoregulatory role rather than a direct bacteria killing activity, as observed in an infection mouse model (122). Recent work using the zebrafish/*M. marinum* model indicates the involvement of macrophage lysosomal CTSs to control the TB infection at the granuloma level (121, 157).

### Granulysin (GNLY)

Granulysin is a small cationic human antimicrobial protein expressed by lymphocytes that is upregulated by HIV/TB coinfection (37, 158). GNLY can enter the macrophages and is able to disrupt the bacillus envelope (7).

### Calgranulin

Calgranulin, also called calprotectin, is another AMP that is used as a TB infection marker in blood samples (30, 124, 127). Calgranulin is a calcium-binding protein that also interacts avidly with Zn^2+^ cations. Binding to Zn^2+^ activates the peptide antimicrobial activity. Recently, calgranulin overexpression has been associated to anti-TB activity at the macrophage intracellular level by promotion of the phagolysosomal fusion (30, 126).

### Ubiquitin-Derived Peptides

Ubiquitin-derived peptides are ubiquitinated proteolytic peptides which can also be classified as AMPs (7, 37, 159). In particular, ubiquitin-conjugated peptides as products of the proteosome degradation activity accumulate in the lysosome and can inhibit Mtb growth within the autophagolysosome (129). Ubiquitin by itself is innocuous while ubiquitinated peptides, such as Ub2, can permeate the mycobacteria membrane (160).

### Human Antimicrobial RNases

Human antimicrobial RNases are small secretory proteins (~15 kDa) belonging to the RNaseA superfamily. They are highly cationic and possess a wide range of biological properties, representing an excellent example of multitasking proteins (112, 161). The family comprises eight human members, expressed in diverse epithelial and blood cell types.

RNase3, also known as the eosinophil cationic protein (ECP), is mainly expressed during infection and inflammation in the secondary granules of eosinophils (162) and secondarily in neutrophils (163). Complementarily, the signal peptide of the ECP (ECPsp) was found to promote the migration of macrophages *via* pro-inflammatory molecules to sites of infection and inflammation (164). Interestingly, ECP is secreted, together with α-defensin, in response to *M. bovis* BCG infection (79). Although the recruitment of eosinophils in the respiratory tract during Mtb infection was first regarded as a mere response to inflammation (165), further work has shown that this cell type together with neutrophils can directly participate in the removal of the infection focus (166). Eosinophils are activated *via* TLR2 induction by the specific mycobacterial wall component, the lipomannan (79). Eosinophils, together with neutrophils, would then release the content of their granules into the granuloma macrophages (84, 159). To note, the *eosinophil peroxidase*, another eosinophil protein stored in the secondary granules, is also endowed with antimycobacterial activity (119). On the other hand, macrophages express upon bacterial infection two additional RNases, RNase6 and RNase7 (114). In addition, human RNase7, also called the skin derived RNase, is also secreted by keratinocytes and exerts a protective role against a variety of pathogens at the skin barrier (39, 115). Interestingly, RNase7, together with RNase3, can eradicate mycobacteria *in vitro* (117). Moreover, very recent results indicate that human RNases 3, 6, and 7 can also inhibit the growth of mycobacteria in a macrophage infection model (167). Considering that RNase6 and RNase 7 expression is induced in macrophages upon bacterial infection (114), one might hypothesize that these antimicrobial proteins can also play a physiological role against intracellular dwelling mycobacteria. Eventually, we cannot disregard a complementary contribution of the RNases reported immunomodulatory properties, such as the induction of pro-inflammatory cytokines and the dendritic cell chemoattraction (168, 169).

## Synthetic Antimicrobial Peptides

In the race against TB, novel synthetic AMPs with potent mycobactericidal activities have been developed (2, 19, 22, 37, 170). AMP synthetic analogs are often considered to be the next generation of antibiotics and have attracted the attention of many companies aiming to develop new anti-TB therapies against drug-resistant strains (35). Following, we summarize the main SAMPs successfully designed (Table 2).

**Table 2:** Synthetic peptides effective against mycobacteria.

Peptide	Modifications	Source	Mechanism/antimicrobial activity	Reference
1-C13_4mer_	Tetrameric form; oligo-N-substituted glycines (peptoid) and alkylation	Design *de novo*	Pore formation MIC (Mtb H37Rv): 6.6 µM	(171)
A18G5, A24C1ac, A29C5FA, and A38A1guan	d-enantiomer, alkylation, tetramethylguanidinilation, and polyethylene glycol conjugation	Derived from the insect proline-rich peptide Apidaecin	Bacterial membrane permeation/inhibition of protein synthesis	Hoffmann R, Czihal P Patent WO2009013262 A1. 2009 (172)
CAMP/PL-D	–	Short cationic peptides (10 AA) rich in W and R selected from peptide libraries	Pore formation MIC (Mtb H37Rv): 1.1–141 µM	(173)
CP26	–	Derived from cecropin A: mellitin	Bacterial cell wall disruption MIC (Mtb H37Rv): 2 µg/mL	(174)
d-LAK 120	d-enantiomer	Synthetic α-helical peptides	Pore-formation/Inhibition of protein synthesis MIC (Mtb H37Rv): 35.2–200 µg/mL	(175, 176)
d-LL37	d-enantiomer	Derived from LL-37	Pore-formation/Immunomodulatory activity MIC (H37Rv): 100 µg/mL	(170)
E2 and E6	–	Derived from bactenecin (bovine cathelicidin) Bac8c (8 AA)	Bacterial cell wall disruption MIC (Mtb H37Rv): 2–3 µg/mL	(174, 177)
HHC-10	–	Derived from bactenecin	Bacteria membrane lysis MIC (*M. bovis* BCG): 100 µg/mL	(178^a^, 179)
hLFcin1-11/hLFcin17-30	d-enantiomer	Derived from lactoferricin (All-R and All-K substitutions)	Bacterial cell wall and membrane lysis IC90 (*M. avium*): 15–30 μM	(44)
Innate defense regulators [innate defense regulator (IDR)-1002, -HH2, IDR-1018]	–	Derived from macrophage chemotactic protein-1 (MCP-1)	Immunomodulatory activity/anti-inflammatory MIC (Mtb H37Rv): 15–30 µg/mL; *in vivo* [Mtb H37Rv and multidrug resistant TB strain (MDR-TB) infected mice]: 10–71% killing at 32 μg/mouse (3 × week intra-tracheal administration, 30 days)	(180)^a^(181)^a^(182)
LLAP		Derived from LL-37	Inhibition of ATPase MIC (*M. smegmatis* mc^2^155): 600 µg/mL	(183)
LLKKK18	Hyaluronic acid nanogel conjugation	Derived from LL-37	Pore formation/Immunomodulatory activity *In vivo* (Mtb H37Rv-infected mice): 1.2-log reduction at 100 µM (10 intra-tracheal administrations)	(184)^a^
MU1140		Derived from *Streptococcus mutans* lantibiotics	Inhibition of cell wall synthesis/On preclinical stage. Effective on active and dormant Mtb MDR	Oragenics Inc Patent WO2013130349A (185)^a^
MIAP	–	Derived from Magainin-I	Inhibition of ATPase MIC (H37Ra): 300 µg/mL	(46)
Pin2 variants		Derived from pandinin2 (short helical peptides)	Membrane disruption Mtb H37Rv and Mtb MDR: 6–14 µg/mL	(186)
RN3(1-45) RN6(1-45) RN7(1-45)	–	Derived from human RNases N-terminus	Bacterial cell wall disruption/cell agglutination and intracellular macrophage killing MIC (*M. vacae; M. aurum; M. smegmatis* mc^2^155*; M bovis* BCG) *in vitro*: 10–20 µM and *ex vivo* (*M. aurum*): 5–10 µM	(117, 167)
Synthetic AMPs (SAMPs-Dma)	Dimethylamination and imidazolation	Design *de novo*	Cell penetration and DNA binding/ synthetic antimicrobial peptide-Dma10: MIC *(M. smegmatis* mc^2^155): <20 µM	(51)
X(LLKK) 2X: II-D, II-Orn, IIDab, and IIDap	Peptide d-enantiomer, ornithination, 2,4-diaminobutyric acidation, and 2,3-diaminopropionic acidation	Short stabilized α-helix amphipatic peptides	Pore formation M(LLKK)2M: MIC (Mtb H37Rv): 125 µg/mL; I(LLKK)2I: effective against MDR-TB	(22, 187)

*^a^Reported activities tested in vivo using murine infection models*.

One of the favorite applied strategies for the design of potent AMPs is the engineering of stabilized amphipathic α-helix that are enriched with selected antimicrobial prone amino acids. Complementarily, peptide modifications are devised to endow them with enhanced resistance to proteolysis; thereby improving their *in vivo* stability and efficacy. The *d-LAK peptides* are a family of serial peptides consisting of 25 d-enantiomer amino acid residues in a primary sequence designed to adopt a left-handed α-helix conformation and containing eight lysine residues (175). The peptides were designed to enhance their antimicrobial activity and decrease their hemolytic effect (188), providing efficient antimycobacterial activity at non-toxic concentrations. Furthermore, d-LAK peptides can be administered as inhalable dry powder (176). Another synthetic α-helical peptide, the M(LLKK)2M, was proven successful against MDR strains when combined with RIF (187). On the other hand, a short synthetic cathelicidin variant (the *HHC-10*) is able to inhibit the growth of *M. bovis* BCG both *in vitro* and in a mouse model (178).

Interestingly, the N-terminus derived peptides of human antimicrobial RNases can reproduce the parental protein activity against several tested *Mycobacterium* species (117, 167). The *RN(1-45)* peptides encompass a highly cationic and amphipathic region that adopts an extended α-helix in a membrane-like environment (189). In addition, the RN3(1-45) and RN6(1-45) peptides include an aggregation prone sequence which promotes bacterial cell agglutination (117, 167, 190), a property that can facilitate the microbial clearance at the infectious focus (190).

Recently, particular interest has been drawn by a collection of short synthetic peptides with immunomodulatory activities, the *innate defense regulators* (IDRs). The peptides are effective at very low concentration and thereby can elude any toxicity to the host (181). They do not display a direct bactericidal activity but can promote the proper endogenous expression of antimicrobial agents by the host cells. Among others, the peptides enhance the release of chemokines and downregulate the inflammation pathway (181, 182). The IDR peptides, such as the IDR-1018 (Table 2), have been tested successfully in a MDR-TB infected mouse model by intra-tracheal administration (180). Likely, immunoregulatory peptides will take a leading role in the treatment of immunocompromised patients in a near future (16).

## AMPs to Combat Antimicrobial Resistance in TB: A Time for Hope

In recent years, thousands of antimicrobial peptides have been identified from natural sources, mostly classified as key players of the non-specific host defense response (30, 33, 191). On the other hand, despite the existence of a wide range of successful antibiotics since their entry into the worldwide trade, nowadays there is an increasing demand of novel drugs to tackle multidrug-resistance mycobacteria strains (2, 20, 192). The antimicrobial proteins and peptides (AMPPs), given their direct bacilli killing mechanism and immunomodulatory properties provide an attractive pharmacological potential against mycobacterial infections (see Table 3 for a summary of main AMP-based therapies). However, despite their appealing properties, AMPs are still facing major challenges to join the pharmaceutical industry (30–32). The main advantages and disadvantages associated with AMPs are listed in Table 4. Although the high cost of synthesis is one of the main drawbacks that the manufacturing of peptides faces, some companies are already managing commercial-scale peptide production platforms. For example, recombinant AMPs can be prepared in fungi and plants at high yield and low cost (2). Another drawback of AMPs therapy is their susceptibility to proteolytic cleavage, in particular when delivered by systemic administration (2, 31). In addition, the antimicrobial activity of some peptides appears to be decimated in physiological saline and serum conditions (32, 193). Novel design strategies are focusing on the production of cheaper and reduced-size analogs (2, 194) with improved selectivity toward prokaryotic targets and broaden therapeutic indexes (195). To improve the peptide bioavailability and stability *in vivo* several strategies have been developed such as incorporation of non-natural amino acids, backbone mimetics, conjugation with fatty acids, N and C- terminus modifications (196). The peptide performance can also be improved by intra-tracheal administration (184). In addition, encapsulation within biodegradable particles or liposomes improves the distribution of the drug toward the site of action (31, 196). Fortunately, macrophage nature by itself should promote the engulfment of such nanovehicles (19) and extensive research has been applied to define the parameters that determine the nanoparticles uptake by the phagocytic cells and intracellular traffic (2, 197). Very recently, a novel delivery system has been achieved by a LL-37 analog embedded within a hyaluronic nanogel. The self-assembled polymer stabilizes the peptide inside its hydrophobic core, allows a higher dose cargo and promotes the macrophage uptake, with increased antimicrobial efficiency and reduced toxicity to host cells. Besides, AMPs are also prone to aggregate and show occasionally poor solubility (198, 199). Luckily, there are currently different strategies and predictive software available to prevent aggregation and improve physicochemical properties (42, 200, 201). Complementarily, cleavage protection can be enhanced through secondary structure stabilization (37, 202). Alternatively, AMPs, as effector players of the host immune system (203) can also be upregulated by immunostimulation therapies (23, 204–206), overcoming the drawbacks inherent to the peptide administration *via*. Furthermore, recent studies on mycobacterial infection reveal how some species such as Mtb are capable of inhibiting the expression and release of endogenous AMPs (30, 77). Thus, the administration of supplementary AMPs, the use of gene therapy (136) or an immunomodulatory hormonal induction would be necessary to achieve an effective dose (207). This approach would be mostly recommended for immunocompromised patients (60, 204, 205). However, researchers should not disregard the unpredictable long-term consequences of exposing the bacteria to an overdose of AMPs. Mycobacteria pathogens exposed to either externally administered or endogenous overexpressed AMPs might develop novel resistance strategies to face back this new affront (2, 146, 208). In fact, the co-evolution of the pathogens with natural AMPs has already induced some bacterial resistance mechanisms (209–212). First, bacteria can alter their cell envelope composition to reduce their affinity toward cationic peptides (82, 209, 212, 213). Pathogenic mycobacteria can also ensure their intracellular survival by the control of the macrophage efflux pump (210, 211). Other observed strategies are the release of extracellular proteases (212, 214, 215) or the downregulation of host AMPs (74). Therefore, resistance to AMPs should be anticipated and might be overcome by innovative peptide variants (216), host-directed therapies or the use of combined synergies (47, 215). Eventually, recent collaborative initiatives have been launched to join efforts in the fight against mycobacterial resistance (TBNET, FightTB, TB-Platform, and TB-PACTS) (2), opening a window of hope.

**Table 3:** AMPs based strategies to develop novel anti-TB drugs.

Pro-autophagy AMPs^a^	Cathelicidins (56, 58, 78); azurocidin (104); calgranulin (126)
Anti-inflammatory AMPs	Defensins (23, 82); AMP binders to antigenic molecules (23); LL-37 inhibition of TNF-α and other pro-inflammatory cytokines (57); synthetic innate defense regulator (IDR) peptides (181); synthetic LLKKK18 (LL-37 analog) (184); lactoferrin (100)
Pro-inflammatory AMPs	LL-37 (57, 74); defensins (82); hepcidin (94)
Chemotaxis induction by AMPs	Defensins (23); IDR synthetic peptides (23); LL-37 (2, 58, 181); elastases (108)
AMP synergy	*with current antibiotics*: HNP1 + isoniazid/rifamicin (141); HBD1 + isoniazid (217); synthetic α-helix AMP + rifampicin (18)
	*with immunomodulators*: HNP1 and HBD2 + l-isoleucine (206)
	*with nanoparticles* (19)
Induction of host AMP expression	Search for novel LL-37 inducers (218); vit D3 and phenylbutirate (PBA) for LL-37 (16, 28, 77, 204); l-isoleucine for β-defensins (83); aroylated phenylenediamine inducers (205)
AMP-based gene therapy	Adenovirus encoding LL-37 or HBD3 (219)
AMP nanodelivery	Nanovehiculation systems: nanoparticle size, surface chemistry, and mechanical properties to enhance macrophage uptake (2); liposomes (2, 196); nanogels (184, 196); aerosolization (176, 196)

*^a^Representative examples are provided for each indicated strategy*.

**Table 4:** Human anti-TB AMP therapy: advantages and disadvantages.

Application strategy	Advantages	Disadvantages
Exogenous AMP administration	Broad-spectrum activity Multi-functionality Low immunogenicity Rapid direct killing mechanism High affinity toward mycobacterial surface Enhanced uptake by macrophages Very low/none toxicity of natural human AMP Rapid clearance in host tissues Beneficial effects to the host (anti-inflammatory, pro-autophagy, anti-tumoral, etc.) Low rate of bacterial resistance emergence High stability and efficacy of modified peptide derivatives Reduced manufacturing cost by new recombinant methodologies Gene therapy can restore endogenous AMPs levels in immunocompromised patients Synergy with current antibiotics Potential use as antibiotic adjuvants	Rapid degradation following oral/systemic administration Low stability in human biological fluids Potential undesirable side-effects at high concentration (tumorigenesis, angiogenesis, etc) Potential toxicity *via* oral/systemic administration High cost of chemical synthesis
Endogenous AMP induction	Efficient at very low concentrations Reinforcement of the immune response in immunocompromised patients Prevention of latent mycobacterial reactivation	No current information on the long-term effects of endogenous AMP induction. Potential induction of AMP resistance

## Concluding Remarks

Peptide-based therapy to treat infectious diseases is recently experiencing resurgence. AMPs, as mere components of the immune system, promote the direct killing of mycobacteria and often have immunomodulatory effects. Their non-specific pleiotropic mechanisms of action and unique immunomodulatory properties over conventional antibiotics have awakened the pharmaceutical market interest. Moreover, the efficacy of BCG vaccine is highly variable and the alarming increase of extensively drug-resistant strains of Mtb is a major global health emergency to address. In this context and considering the limitations in the current antituberculosis drug treatment, AMPs represent an immediate alternative approach in tackling antimicrobial resistance. Scientific evidences provide a solid basis to ensure that the future development of peptide-based therapy will continue to address the unsolved drawbacks that the pharmaceutical industry is currently facing. Novel research methodologies and integrated interdisciplinary strategies should provide the opportunity to boast current antimicrobial peptide research efforts in the fight against tuberculosis.

## Author Contributions

JA-T, LL, DP, SB, and EB contributed to the original draft and edited versions. JA-T and LL prepared the graphical material and JA-T, DP, LL, and EB prepared the tables. SB and EB wrote, edited, and revised the final manuscript version. All authors approved the final manuscript version.

## Conflict of Interest Statement

No potential conflicts of interest were disclosed. The authors declare that the research was conducted in the absence of any commercial or financial relationships that could be construed as a potential conflict of interest.
